# A Comparative Study of Two Models of Intraluminal Filament Middle Cerebral Artery Occlusion in Rats: Long-Lasting Accumulation of Corticosterone and Interleukins in the Hippocampus and Frontal Cortex in Koizumi Model

**DOI:** 10.3390/biomedicines10123119

**Published:** 2022-12-02

**Authors:** Mikhail V. Onufriev, Mikhail Y. Stepanichev, Yulia V. Moiseeva, Marina Y. Zhanina, Olga A. Nedogreeva, Pavel A. Kostryukov, Natalia A. Lazareva, Natalia V. Gulyaeva

**Affiliations:** 1 Laboratory of Functional Biochemistry of the Nervous System, Institute of Higher Nervous Activity and Neurophysiology, Russian Academy of Sciences, 117485 Moscow, Russia; 2 Research and Clinical Center for Neuropsychiatry of Moscow Healthcare Department, 115419 Moscow, Russia

**Keywords:** stroke, middle cerebral artery occlusion, hippocampus, frontal cortex, distant damage, corticosterone, interleukins, neuroinflammation, HPA axis

## Abstract

Recently, we have shown the differences in the early response of corticosterone and inflammatory cytokines in the hippocampus and frontal cortex (FC) of rats with middle cerebral artery occlusion (MCAO), according to the methods of Longa et al. (LM) and Koizumi et al. (KM) which were used as alternatives in preclinical studies to induce stroke in rodents. In the present study, corticosterone and proinflammatory cytokines were assessed 3 months after MCAO. The most relevant changes detected during the first days after MCAO became even more obvious after 3 months. In particular, the MCAO-KM (but not the MCAO-LM) group showed significant accumulation of corticosterone and IL1β in both the ipsilateral and contralateral hippocampus and FC. An accumulation of TNFα was detected in the ipsilateral hippocampus and FC in the MCAO-KM group. Thus, unlike the MCAO-LM, the MCAO-KM may predispose the hippocampus and FC of rats to long-lasting bilateral corticosterone-dependent distant neuroinflammatory damage. Unexpectedly, only the MCAO-LM rats demonstrated some memory deficit in a one-trial step-through passive avoidance test. The differences between the two MCAO models, particularly associated with the long-lasting increase in glucocorticoid and proinflammatory cytokine accumulation in the limbic structures in the MCAO-KM, should be considered in the planning of preclinical experiments, and the interpretation and translation of received results.

## 1. Introduction

Ischemic stroke, the more common type of stroke, is a severe neurological disease and a global leading cause of death and disability [1]. Delayed post-stroke consequences are quite frequent and include both neurological and cognitive disorders [2]. Stroke therapy and the prevention of its delayed consequences are one of the hottest areas in preclinical studies; however, advancements in treating stroke and its sequelae appear much slower than expected. It is no secret that during recent decades the candidate neuroprotective drugs showing the most promising results in routine rodent models of ischemic stroke have failed in clinical trials [3,4,5]. One of the main reasons for this continuous failure may be due to problems with the validity of the models used [6,7]. Though the involvement of the stress and neurohumoral system in strokes is well-documented in clinical studies, it is still neglected in rodent models of ischemic stroke [8], although increased cortisol is associated with a worse outcome and the development of delayed cognitive deficits. Limbic structures, primarily the hippocampus, which coordinate both cognitive and emotional functions, have a high density of corticosteroid receptors, and this may be related to their selective vulnerability to stress factors. It was hypothesized that glucocorticoid-dependent hippocampal damage and dysfunction may be associated with the cognitive and emotional consequences of ischemic stress [9,10].

Occlusion of the middle cerebral artery (MCAO) in rodents with an intraluminal filament is regarded as a golden standard to model ischemic stroke. Among the most popular approaches, the two classical surgical methods of Koizumi et al. [11] and Longa et al. [12] are randomly used in preclinical studies; most authors analyze the data received using either model without taking into account that, in fact, they are not identical. Comparisons of these MCAO models in mice demonstrated significant dissimilarities between them along with an obvious resemblance [13,14]. Recently, we have performed a direct comparison of the Koizumi model (MCAO-KM) and Longa model (MCAO-LM) in rats [15]. Though the infarct volume, mortality rate, neurological deficits, and weight loss were similar in the MCAO-KM and MCAO-LM rats, during the acute period, the MCAO-KM animals showed elevated corticosterone (CS) and interleukin-1β (IL1β) in blood serum. CS accumulation was shown in the ipsilateral and contralateral frontal cortex (FC) and the hippocampus of the MCAO-KM group, whereas IL1 β was specifically elevated in the ipsilateral hippocampus in the MCAO-KM rats. Thus, the MCAO-KM rats may be predisposed to CS-dependent distant neuroinflammatory hippocampal damage.

The aim of the present study was to assess the delayed effects of the MCAO-KM and MCAO-LM in rats on the CS and proinflammatory cytokines in blood, the hippocampus, and FC 3 months after MCAO. We also performed a behavioral screening of these animals to test for potential changes in their cognitive and emotional spheres.

## 2. Materials and Methods

### 2.1. Environment and Housing

The study was performed on male 3-month-old Wistar rats (BW 200–300 g at the beginning of the study). The animals were purchased from the “Stolbovaya” Breeding Center (Moscow Region, Russia) and were housed in acrylic cages, with spruce shavings provided as bedding, in the institutional vivarium. The rats were kept in a 12 h:12 h day cycle, with free access to food and water. All experiments with animals were performed in accordance with the EU Directive 2010/63/EU. The experimental protocol was approved by the Ethical Commission of the Institute of Higher Nervous Activity and Neurophysiology, Russian Academy of Sciences (protocol number 10, 10 December 2012). The ARRIVE Guidelines (v. 2.0) were followed. All efforts were made to minimize animal suffering. The number of animals was calculated from the pilot series of experiments with the power goal set as 0.8. For the behavioral experiments, the minimum number of animals per group was 7. The rats were numbered before starting the experimental session and randomly assigned to 4 groups using a random number generator prior to the experimental Day 0: MCAO was performed using the Koizumi method (MCAO-KM) [11]; sham-operated using Koizumi method (SHAM-KM); MCAO was performed using the Longa method [12] (MCAO-LM); sham-operated using Longa method (SHAM-LM). Due to the rather high mortality rate, the final groups contained different number of animals: MCAO-KM, n = 8; SHAM-KM, n = 13; MCAO-LM, n = 9; SHAM-LM, n = 10.

### 2.2. Modeling Ischemic Stroke

#### 2.2.1. MCAO-KM

The surgery was performed according to the method of Koizumi et al. [11]. Rats were anesthetized with isoflurane. An incision was made in the neck area and, pushing the muscle tissue on the left side, penetration to the common carotid artery was performed, and ligatures were applied to it, as well as to the external and internal carotid arteries. A nylon filament (3–0) with a rounded end was inserted through the hole at the bifurcation site onto the external and internal branches and advanced along the internal carotid artery to the middle cerebral artery. Then, the ligature on the internal carotid artery was tightened to fix the filament. The occlusion lasted for 60 min; common, external, and internal carotid arteries remained ligated, and the body temperature of the animal was maintained at 37 ± 0.5 °C. Then, the filament was removed, and the ligature on the internal carotid artery was tightened. Thus, after occlusion, the common, external, and internal carotid arteries on the left side remained ligated, and reperfusion occurred mainly at the expense of the Willis circle. In the SHAM-KM group, all these manipulations were performed, except for the introduction of the filament.

#### 2.2.2. MCAO-LM

The surgery was performed according to the method proposed by Longa et al. [12] under isoflurane anesthesia. Through an incision in the neck of the animal, the common carotid and external carotid arteries were assessed and ligated. After electrocoagulation and dissection of a fragment of the left external carotid artery near the bifurcation, a filament was inserted through the remaining part of the artery and pushed through the internal carotid artery to the intersection with the middle cerebral artery. The occlusion lasted for 60 min, while the body temperature of the rat was maintained in the range of 37 ± 0.5 °C. Then, the filament was extracted, and blood flow was restored along the ipsilateral common carotid artery, which was released from the ligature after the extraction of the filament. In the SHAM-LM group, all these manipulations were performed, except of the introduction of the filament.

Rats in the MCAO groups without signs of lesion—paresis of the contralateral foreleg (score 1–2 according to the 5-point scale) or those with maximal deficit (score 5, unresponsive), were excluded from the study. No sham animals died or showed signs of lesions or significant neurological deficits.

### 2.3. Assessment of Neurological Deficits

#### 2.3.1. Five-Point Neurological Scale

Motor and behavioral changes were assessed using a 0–5-point grading scale [16] at 1 h following MCA occlusion and daily prior to sacrifice. This standard test, used conventionally to assess the efficiency of focal brain ischemia in rodents, is based on a 5-point behavioral scale and allows the evaluatation of the functional state of the contralateral foreleg of the rat, the presence of turns and circulation in the contralateral side, as well as the mobility of the animal: 0, no deficit; 1. failure to extend right forepaw fully; 2. decreased grip of right forelimb while tail pulled; 3. spontaneous circling or walking to contralateral side; 4. walks only when stimulated with depressed level of consciousness; 5. unresponsive to stimulation.

#### 2.3.2. Tongue Protrusion Test

This test allows the assessment of the ability of the rat to lick peanut butter out from a thin, full glass cylinder left in the cage overnight [17]. The length of the butter pile, from the beginning of the cylinder to the level of the remaining butter, shows the ability of the animal to protrude its tongue, which is impaired during the acute phase of MCAO.

### 2.4. Behavioral Studies

The behavior of animals from 4 groups was studied 3 months after MCAO or sham surgery. All tests except for the sucrose preference test were performed between 11.00 and 14.00, whereas the sucrose preference was assessed between 17.00 and 19.00. For this purpose, the rats were transported from the colony room to the experimental room at least 1 h prior to testing. Animal behavior was recorded using a DMK 23GV024 GigE video camera and IC Capture, v. 2.2 software (both The Imaging Source Europe, GmbH, Bremen, Germany) and analyzed, as a rule, using EthoVision XT 11 software (Noldus, Wageningen, The Netherlands). All devices for behavioral studies were supplied by Open Science, Ltd. (Moscow, Russia). All tests were performed by two investigators blinded to the initial distribution of animals between the groups.

#### 2.4.1. Open Field Test (OFT)

OFT was used to assess locomotor and exploratory activity in rats. The animals were tested in a round arena with a diameter of 100 cm and surrounded by a 30 cm wall. The rat was placed into the center of the arena for 5 min. The distance traveled, rearing, entries to the center of the arena, and defecations were counted.

#### 2.4.2. Elevated Plus Maze (EPM)

The EPM was made of plastic-covered plywood and consisted of two opposed open arms (50 × 10 cm), two opposed enclosed arms (50 × 10 × 40 cm) and an open square (10 × 10 cm) in the center. The maze was elevated 70 cm above the floor. The animals were placed into the central square of the maze facing one of the open arms for 5 min. The number of entries into and the time spent in the open and enclosed arms were registered visually.

#### 2.4.3. Forced Swim Test (FST)

FST was performed in a cylinder with a diameter of 20 cm and a height of 60 cm, containing 40 cm of clean water (22 °C). The cylinder was thoroughly cleaned, and water was changed between rats. After the test, the animals were carefully dried and kept under a heating fan for 1 h before returning to their home cages. On Day 1, the rats were subjected to a single 15 min swimming session. On Day 2, a second 5 min swimming session was conducted. Duration of immobility, i.e., episodes when the rats remained motionless or floating, including subtle movements to keep their heads above the water, was recorded.

#### 2.4.4. Sucrose Consumption and Preference Tests

Sucrose intake is a measure of hedonia, and anhedonia, a decreased sucrose consumption (or preference), is considered to be an index of depressive-like state in animals. Each rat was placed in a test cage identical to the home cage, and the fluid intake (consumption of 20% sucrose solution) was recorded for 15 min [18]. The bottles with sucrose solution were weighed prior to and after the testing, and the difference was considered as a measure of consumption. The animals were neither food- nor water-deprived prior to testing. The rats were pre-exposed to the sucrose consumption test for 4 days (adaptation to the procedure), and the values of fluid intake on Day 5 were used for statistical evaluation of differences between groups of rats. Twenty-four hours after the last sucrose consumption test, the sucrose preference test was performed. The animals were exposed to both the test solution (20% sucrose) and drinking water for 1 h following 23 h of food and water deprivation. Sucrose preference was calculated as a ratio of sucrose intake (g), and the total liquid consumption (g) was expressed as a per cent.

#### 2.4.5. Spontaneous Alternation Behavior in a Y-Maze

This test was used to assay short-term (working) memory in rats. Spontaneous alternation behavior is based on the ability of animals to enter an arm of a Y-maze not entered in the previous choices [19]. The maze consisted of three arms (42.5 × 14.5 × 22.5 cm) connected to each other under an angle of 120°. The floor of each arm was covered by sawdust, which was changed between trials. The rat was placed into one of the arms of the maze and allowed to explore it for 8 min. The series of arm entries, including returns into the same arm, were recorded. An alternation was defined as entries into all three arms on consecutive occasions. The number of maximum alternations was, therefore, the total number of arm entries minus two, and the percentage of alternation was calculated as (actual alternations/maximum alternations) × 100. 

#### 2.4.6. Step-Through Passive Avoidance

Two days after the end of the sucrose preference test, the animals were trained in a one-trial step-through passive avoidance task to estimate learning and memory. The Panlab Shuttle box (Panlab, Barcelona, Spain) consisting of two equally sized compartments with two independent grid floors was used for training. One compartment was illuminated (700 lx), and the other one was dark (2 lx). On the first day of the passive avoidance training, each rat was placed into the light compartment, and a sliding door was opened 30 s later, allowing the rat to go into the dark compartment. The step-through latency was recorded when the rat stepped into the dark compartment with all four paws, and the sliding door was closed after that. This procedure was repeated twice with the inter-trial interval of 30 min. Then, the rat was placed into the light compartment for the third time. As soon as the rat entered the dark compartment, scrambled foot shock was applied to the grid floor (0.6 mA, 5 s). After a further 30 s, the rat was removed from the experimental box and returned to its home cage. Retention of the aversive training experience was tested 24 h after the training. The cut-off time of 300 s was set for the step-through latency during the retention trial.

### 2.5. Collection of Biomaterial

After the end of behavioral studies, the animals were instantly decapitated, and the following biological material was obtained: post-decapitation blood; and the ipsi- and contralateral regions of the brain—the hippocampus (whole structure from each hemisphere) and frontal cortex (FC, +6.1–3.0 mm from bregma). Subsequently, the blood was centrifuged 1500× *g* at 40 °C for 15 min to obtain serum. The isolated brain regions were homogenized in a Potter homogenizer using a 10-fold excess of a cold standard phosphate–salt buffer containing 0.1% nonidet P-40(Roche) and a cocktail of protease inhibitors (Thermo Scientific, Waltham, MA, USA) with 10 impacts of the pestle at a rotation speed of 1000 rpm. The homogenates were centrifuged at 13,000× *g* at 40 °C for 30 min to obtain a soluble protein fraction (supernatant), which was aliquoted and stored at −80 °C before biochemical studies. The samples were recoded for the following biochemical assay.

### 2.6. Enzyme-Linked Immunosorbent Assays (ELISA)

To determine the serum and brain tissue corticosterone levels, kits for the enzyme-linked immunosorbent assay (Kit Corticosterone for 96 tests, cat. no. EIA4164; DRG, Germany) were used; the kits allow the detection of both free and transport protein-bound corticosterone using a competitive ELISA method. Serum adrenocorticotropic hormone (ACTH) levels were assessed using the ACTH (Rat, Mouse) Extraction Free EIA Kit (EKE-001-21, Phoenix Pharmaceuticals, Burlingame, CA, USA) according to the manufacturer’s instructions. The levels of proinflammatory cytokines interleukin 1β (IL1β), interleukin 6 (IL6), and tumor necrosis factor α (TNFα) in blood serum and brain tissue of rats were measured using R&D Systems Quantikine ELISA Kits (Minneapolis, MN, USA) according to the manufacturer’s instructions (cat.no. SRLB00; cat. No. SR6000B; cat. No. SRTA00).

**Figure 1:**
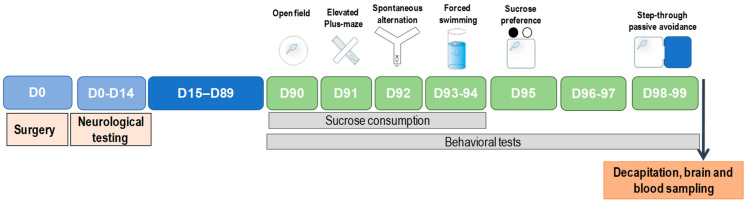
Experimental design.

The general scheme of experimental design of the study is presented in Figure 1.

### 2.7. Statistical Analysis

After the data accumulation, descriptive statistics were calculated, including means, medians, standard deviations, standard errors of mean, and lower and upper quartiles. The data were checked for normality using the Shapiro–Wilk W test. For statistical analysis of the data on neurological deficits after MCAO assessed using the 5-point scale and tongue protrusion test, repeated measures ANOVA and multiple comparisons were performed using posthoc Tukey HSD test. For the analysis of biochemical data, factorial ANOVA followed by posthoc Tukey HSD tests were used. Comparison of behavioral data was performed using Student’s *t*-test with correction for multiple comparisons or Mann–Whitney U test depending on the normality of data distribution because the data did not pass Levene’s test for variance homogeneity. The results are presented as individual points and the mean ± SEM. The differences between groups were considered significantly different at *p* < 0.05 and as a trend at *p* < 0.1.

## 3. Results

### 3.1. Mortality and Neurological Deficits in MCAO-KM and MCAO-LM

For rats showing a neurological deficit according to the 5-point scale, the survival rate within 48 h was 52% (12 of 23) for the MCAO-KM rats and 52% (11 of 21) for the MCAO-LM rats; the survival within 3 months was 35% (8 of 23) and 43% (9 of 21), respectively. The short-term survival did not differ significantly from the data reported previously for the MCAO-KM (57% within 72 h) and the MCAO-LM (63%) [15]. Similar to the reports of Smith et al. [13] and Morris et al. [14], in mice, the mortality did not differ in the MCAO-LM and MCAO-KM and was very high during the first 24 h after MCAO (e.g., the after 60 min occlusion survival rate was 38.5% within 24 h in the MCAO-KM mice). 

**Figure 2:**
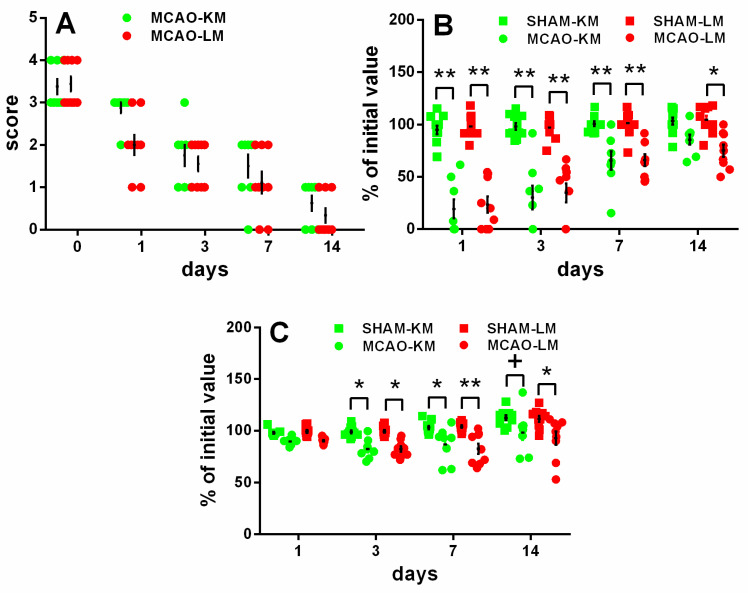
Time course of neurological deficits (**A**,**B**) and body weight loss (**C**) in MCAO-KM, MCAO-LM, and respective sham groups during 14 days after surgery. (**A**) 5-score test after surgery 0, 1, 3, 7, and 14 days after surgery. (**B**) Tongue protrusion test 1, 3, 7, and 14 days after surgery; % of initial values. (**C**) Body mass 1, 3, 7, and 14 days after surgery; % of initial mass. ** *p* < 0.01, * *p* < 0.05, ^+^ *p* < 0.1 compared with the respective sham-operated groups.

The rats in both the MCAO-KM and MCAO-LM groups showed remarkable neurological deficits in the 5-score test (Figure 2A) 1, 3, and 7 days after surgery, whereas almost no deficit was evident at 2 weeks. The neurological deficit according to the 5-score test (Figure 2A) decreased with time within 14 days (ANOVA: F(4,60) = 126.36, *p* = 0.000001); however, it did not depend on the model (F(1,15) = 2.06, *p* = 0.17; MCAO-KM vs. MCAO-LM on Days 0, 1, 3, 7, and 14 was *p* = 1, 0.123, 0.99, 093, and 0.99, respectively). 

The tongue protrusion test results (Figure 2B) showed a significant decrease in both the MCAO groups compared with the respective shams on Days 1, 3, and 7, though this neurological deficit recovered gradually; on Day 14, only a slight decrease could be seen in the MCAO-LM group. The neurological deficit, according to the tongue protrusion test (Figure 2B), significantly decreased with time within 14 days (ANOVA: F(3,108) = 47.52, *p* = 0.000001); however, the time course of the tongue protrusion changes did not depend on the model (ANOVA: F(1,36) = 0.020, *p* = 0.88; MCAO-KM vs. MCAO-LM on Days 0–14, *p* = 0.95–1.00). 

The body mass loss on Days 3 and 7 (Figure 2C) was significant in both MCAO groups, and on Day 14, it was significant in the MCAO-LM group and showed a trend in the MCAO-KM animals (ANOVA: F(1,36) = 35.13, *p* = 0.000001), but it did not depend on the model either (ANOVA: F(1,36) = 0.104 *p* = 0.74; MCAO-KM vs. MCAO-LM, *p* = 0.99–1.0 at all time points).

The lack of difference in the neurological deficits in these two models is important, suggesting that delayed differential biochemical changes are not closely related to the primary MCAO-induced brain damage reflected in the acute neurological deficits. The bilateral changes in the corticosterone accumulation in the hippocampus and FC also confirm the lack of a tight association with the primary unilateral damage. Considering our hypothesis on delayed glucocorticoid-dependent hippocampal damage after focal brain injury [9], these data support our suggestion and are in line with clinical data showing no direct correlation between acute neurological deficits and the development of delayed post-stroke cognitive and emotional disturbances.

### 3.2. Corticosterone and Pro-Inflammatory Interleukins in Blood Serum

The blood levels of corticosterone, ACTH, IL1β, TNFα, and IL6 in the four groups of rats 3 months after surgery are presented in Table S1. None of the parameters differed between the MCAO-KM or MCAO-LM and the respective sham groups 3 months after surgery. The only trend (*p* < 0.1) was revealed in the corticosterone level which tended to be higher in the MCAO-LM compared with the MCAO-KM.

### 3.3. Corticosterone and Pro-Inflammatory Interleukins in the Hippocampus and Frontal Cortex

**Figure 3:**
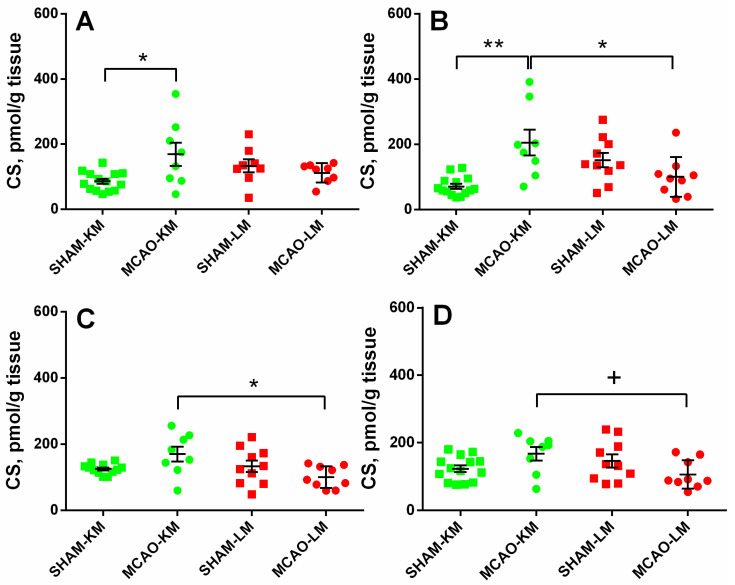
Corticosterone levels in the hippocampus (**A**,**B**) and FC (**C**,**D**) of MCAO-KM and MCAO-LM groups 3 months after surgery. (**A**) Ipsilateral hippocampus. (**B**) Contralateral hippocampus. (**C**) Ipsilateral FC. (**D**) Contralateral FC. (**A**–**D**) Corticosterone (CS), pmol/g tissue.** *p* < 0.01, * *p* < 0.05, and ^+^ *p* < 0.1 compared with respective sham-operated groups or MCAO-KM and MCAO-LM.

The corticosterone level in the ipsilateral hippocampus (Figure 3A) was increased 3 months after MCAO (ANOVA: F(1,36) = 10.18, *p* = 0.002) only in the MCAO-KM group compared with the respective sham group (*p* = 0.048). In the contralateral hippocampus (Figure 3B), the corticosterone level was higher in the MCAO-KM group (ANOVA: F(1,37) = 18.50, *p* = 0.0001) compared with either the respective sham group (*p* = 0.0005) or the MCAO-LM animals (*p* = 0.014). In the ipsilateral FC (Figure 3C), the corticosterone level was higher in the MCAO-KM rats (ANOVA: F(1,37) = 6.80, *p* = 0.013) compared with the MCAO-LM model (*p* = 0.018), whereas in the contralateral FC (Figure 3D) (ANOVA: F(1,37) = 7.52, *p* = 0.009), this difference was expressed as a trend.

**Figure 4:**
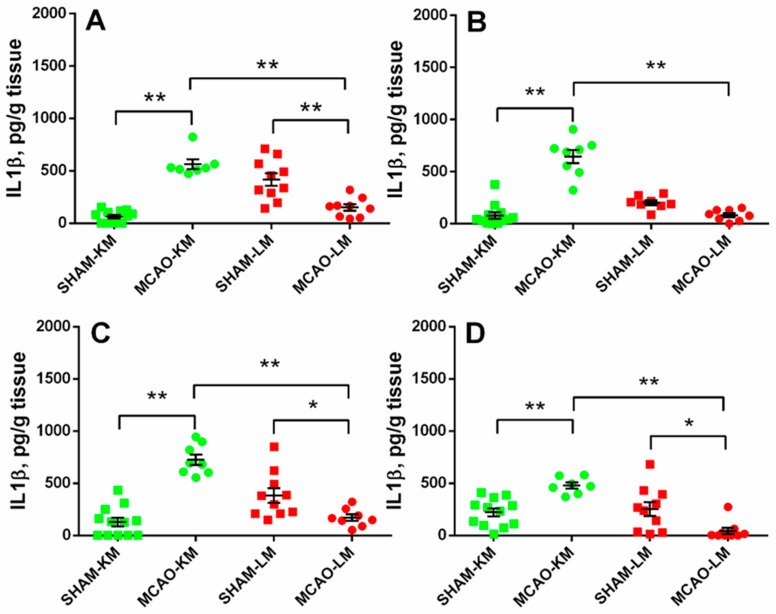
IL1β levels in the hippocampus (**A**,**B**) and FC (**C**,**D**) of MCAO-KM and MCAO-LM groups 3 months after surgery. (**A**) Ipsilateral hippocampus. (**B**) Contralateral hippocampus. (**C**) Ipsilateral FC. (**D**) Contralateral FC. (**A**–**D**) IL1β, pg/g tissue. ** *p* < 0.01 and * *p* < 0.05 compared with the respective sham-operated groups or MCAO-KM and MCAO-LM.

In both the ipsilateral (ANOVA: F(1,34) = 88.10, *p* = 0.000001) and contralateral (ANOVA: F(1,32) = 81.80, *p* = 0.0000001) hippocampus, the IL1β levels in the MCAO-KM group were higher than in the respective shams (*p* = 0.00016 and *p* = 0.00017) and the MCAO-LM (*p* = 0.00016 and *p* = 0.00016) rats (Figure 4A,B). Surprisingly, the IL1β level in the MCAO-LM group was lower than in the respective shams (ipsi Hip, *p* = 0.00038; ipsi FC, *p* = 0.040; contra FC, *p* = 0.015). Similar changes were evident in both the ipsilateral (ANOVA: F(1,34) = 59.55, *p* = 0.000001; MCAO-KM vs. sham, *p* = 0.00016) and contralateral (ANOVA: F(1,34) = 29.19, *p* = 0.000022; MCAO-KM vs. sham, *p* = 0.0036) FC (Figure 4C,D). In the MCAO-LM shams, the IL1β levels were higher in the ipsilateral hippocampus compared with the contralateral (*p* = 0.012; Figure 4A,B), suggesting an acute inflammatory response of the hippocampus from the sham-lesioned side.

**Figure 5:**
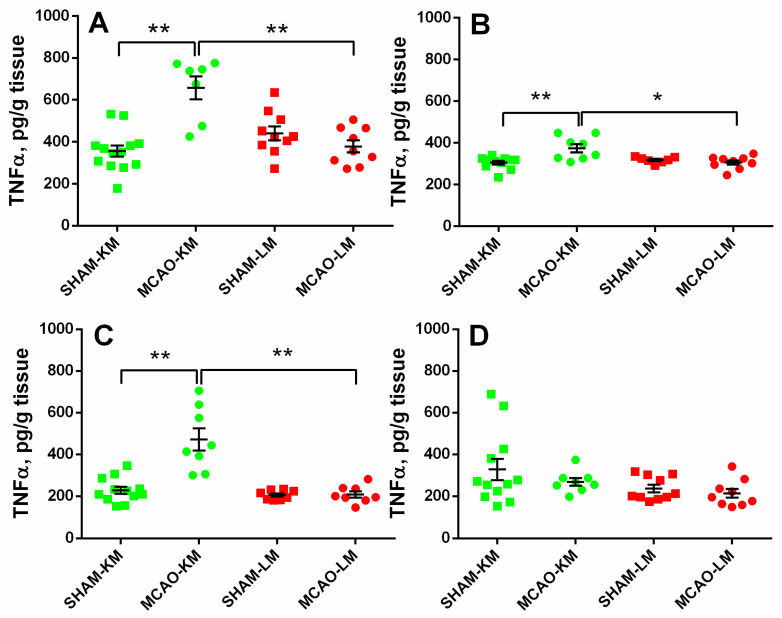
TNFα levels in the hippocampus (**A**,**B**) and FC (**C**,**D**) of MCAO-KM and MCAO-LM groups 3 months after surgery. (**A**) Ipsilateral hippocampus. (**B**) Contralateral hippocampus. (**C**) Ipsilateral FC. (**D**) Contralateral FC. (**A**–**D**) TNFα, pg/g tissue. ** *p* < 0.01 and * *p* < 0.05 compared with the respective sham-operated groups or MCAO-KM and MCAO-LM.

In both the ipsilateral (ANOVA: F(1,35) = 27.28, *p* = 0.000008) and contralateral (ANOVA: F(1,31) = 10.88, *p* = 0.024) hippocampus, the TNFα levels were significantly higher in the MCAO-KM group compared with either the respective shams (*p* = 0.00016 and *p* = 0.0010) or the MCAO-LM (*p* = 0.00020 and *p* = 0.025) group (Figure 5A,B). Similar augmentation in TNFα was also revealed in the ipsilateral FC (ANOVA: F(1,32) = 18.85, *p* = 0.00013; MCAO-KM vs. sham, *p* = 0.00017). However, in the contralateral FC (ANOVA: F(1,35) = 0.27, *p* = 0.60), no differences between the four groups could be demonstrated. 

Three months after the surgery, IL6 levels did not change after MCAO, irrespective of the model used either in the hippocampus or FC in the ipsilateral and contralateral hemispheres (Figure S1A–D).

All results of the biochemical analyses in the hippocampus and FC are summarized in Table S2.

### 3.4. Behavioral Studies

#### 3.4.1. Locomotion and Exploration Behavior Tests

Locomotion and exploration were studied in the OFT. Rats from the MCAO-LM group walked a longer distance within the arena compared with the respective sham-operated animals (*p* < 0.01 according to the Mann–Whitney test; Figure S2A, whereas the distance traveled did not differ between the MCAO-KM and respective shams. Interestingly, the MCAO-LM animals covered a longer distance at the periphery of the arena compared with the respective sham-operated rats (*p* < 0.001), whereas in the central zone of the arena, these animals moved similarly to the sham-operated rats (Figure S2B,C). Rearing, a form of exploratory activity, did not differ in either model studied (Figure S2D–F). Animals in the MCAO-LM group moved with higher velocity than the respective shams (*p* < 0.01; Figure S2G).

#### 3.4.2. Anxiety and Depressive-Like Behavior Tests

The ratio between the distances traveled in the central and peripheral zones of the OFT arena, considered as a measure of anxiety, did not change in the MCAO-KM group but decreased in the MCAO-LM group compared with the respective sham-operated rats (*p* < 0.01; Figure S2H). No changes in the number of defecation boli (Figure S2I), potentially reflecting the emotionality and fear in the animals exposed to a new unfamiliar environment, could be found.

The absence of a significant delayed effect of brain ischemia on the anxiety in rats was further supported by the results of the EPM test (Figure S3). The rats of the MCAO-KM and MCAO-LM groups displayed a similar number of entries into the open or closed arms of the EPM and spent a similar time in those arms as the respective sham-operated animals.

**Figure 6:**
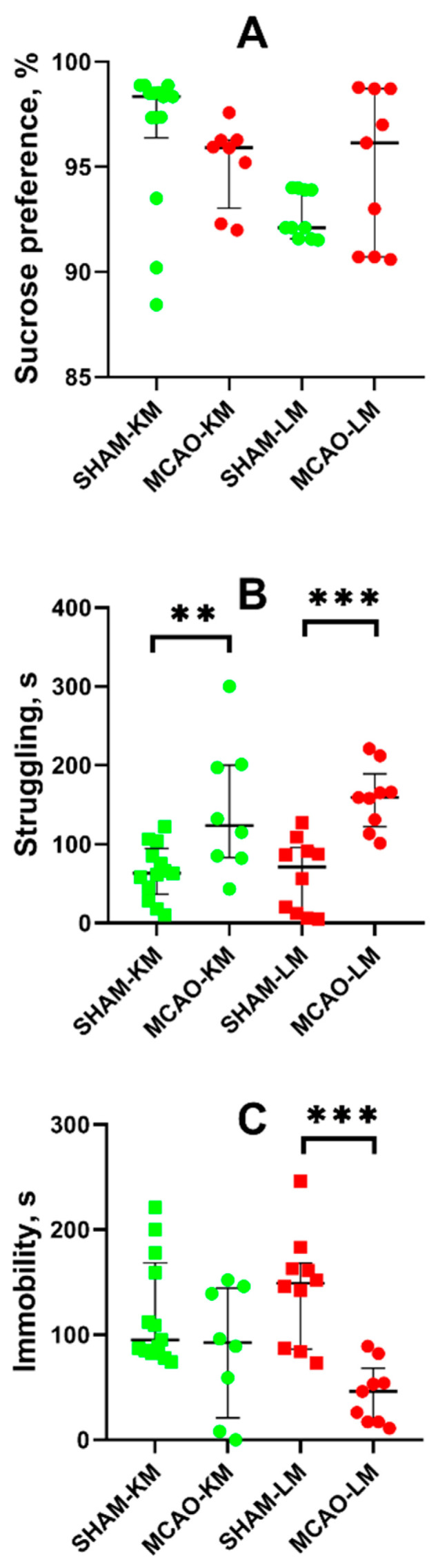
Effects of focal brain ischemia on behavior of MCAO-KM and MCAO-LM groups in the sucrose preference (**A**) and forced swim tests (**B**,**C**) 3 months after surgery. (**A**) Sucrose preference. (**B**) Struggling behavior. (**C**) Immobility duration. ** *p* < 0.01 and *** *p* < 0.005 (Mann–Whitney U test).

Anhedonia, a core feature of depression, was estimated in animals using the sucrose preference test. The MCAO-KM and MCAO-LM rats showed similar levels of sucrose preference as the respective sham-operated rats (Figure 6A).

In the FST, both the MCAO-KM and MCAO-LM rats exhibited a longer period of struggling behavior, i.e., the time before the first immobility episode (*p* < 0.01; Figure 6B). In both MCAO groups, the immobility duration showed a downward trend, although it was significant only in the MCAO-LM compared with the respective shams (*p* < 0.005; Figure 6C). 

#### 3.4.3. Learning and Memory Tests

**Figure 7:**
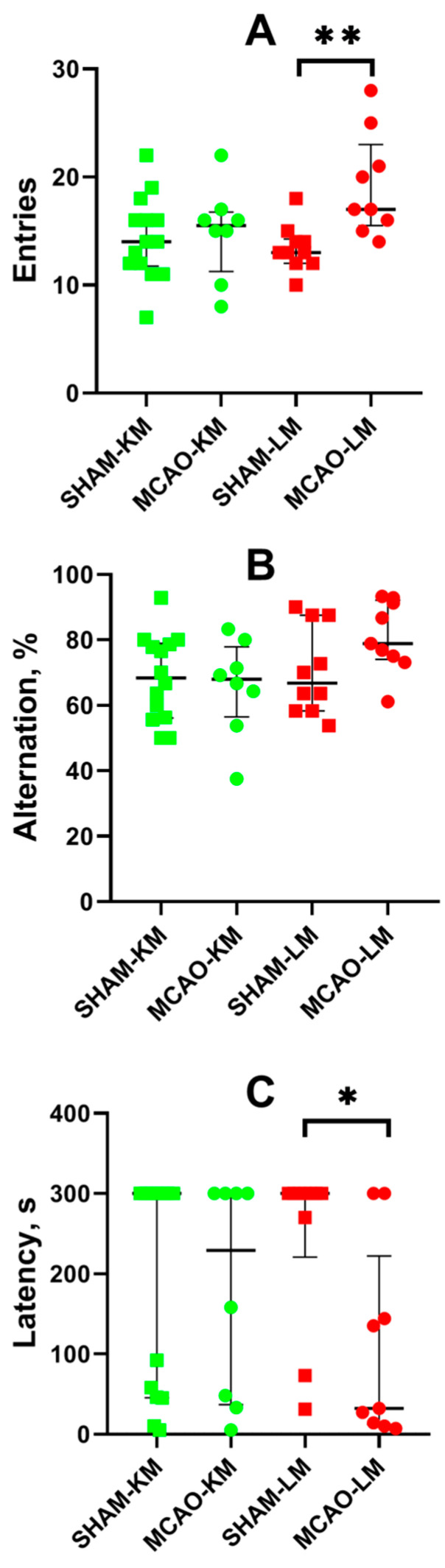
Effects of focal brain ischemia on behavior of MCAO-KM and MCAO-LM groups in the Y-maze (**A**,**B**) and passive avoidance test (**C**) 3 months after surgery. (**A**) Number of entries into the arms of Y-maze. (**B**) Alternation (%). (**C**) Step-through latency in the passive avoidance test, s. * *p* < 0.05 and ** *p* < 0.01 (Mann–Whitney U test).

The spontaneous alternation behavior in the Y-maze revealed more entries into the arms of the maze by the MCAO-LM group rats (but not the MCAO-KM) compared with the sham-operated animals (*p* < 0.01; Figure 7A). This effect is associated with higher locomotor activity in the OFT of the MCAO-LM rats. No difference in the alternation behavior was found in either MCAO group compared with the respective shams (Figure 7B).

Long-term memory was studied using the step-through passive avoidance training. In the pretraining session, the animals entered the dark compartment of the experimental chamber, demonstrating similar step-through latencies (M ± SD: 14.3 ± 13.0, 11.1 ± 9.6, 13.7 ± 6.9, and 11.6 ± 10.3 s in the SHAM-LM, MCAO-LM, SHAM-KM, and MCAO-KM groups, respectively). Twenty-four hours after the training, the animals were put into the light compartment of the chamber again to test their capability to retain memories. No significant decrease in the step-through latency in the MCAO-KM group compared with the SHAM-KM group was found. However, the latency decreased in the MCAO-LM group compared with the respective shams (*p* < 0.05; Figure 7C). 

## 4. Discussion

MCAO, using surgical approaches with intraluminal filaments, remains a golden standard of ischemic stroke modeling in rodents after about 3 decades. At present, two classical models, the MCAO-KM and MCAO-LM, are routinely used as alternatives in experimental preclinical studies. These MCAO models in mice were directly compared and, though similar in many respects, showed significant differences [13,14]. Recently, we have performed a direct comparison of MCAO-KM and MCAO-LM in rats during the early post-ischemic period [15]. We showed a lack of differences in the infarct volume, mortality rate, neurological deficits, and weight loss in these models 3 days after MCAO. However, important differences were found in the HPA axis and pro-inflammatory cytokines in blood and brain regions. Specifically, the MCAO-KM rats demonstrated increases in blood corticosterone and IL1 β, whereas the MCAO-LM rats showed an increase in ACTH levels in blood serum. In the FC and hippocampus of the MCAO-KM group (but not the MCAO-LM), corticosterone and IL1β beta accumulation was found. Thus, 3 days after the surgery, corticosterone and IL1β release, as well as hippocampal accumulation, were more expressed in the MCAO-KM rats, which might predispose MCAO-KM animals to corticosterone-dependent distant neuroinflammatory hippocampal damage. In this study, the delayed effects of these two MCAO models on the HPA axis indices and pro-inflammatory cytokines were studied 3 months after surgery. The data demonstrate that in the MCAO-KM specifically, the accumulation of corticosterone and pro-inflammatory cytokines in the hippocampus and FC can persist for months.

The functioning of the hypothalamic–pituitary–adrenocortical (HPA) axis underlies stress response and adaptation. Glucocorticoids (cortisol in humans, corticosterone in rodents), the executive adrenal hormones of the HPA axis, are major managers of brain plasticity underlying the adaptation of the brain and ensuring its integrative function. Cerebral ischemia, a common stress factor, induces the responses of the HPA axis. Ischemia-induced HPA activation which represents a quick physiological response, resulting in a prolonged increase in blood glucocorticoid levels, was described about 3 decades ago [20,21,22]. The fact that the stress response mediated by the HPA axis is associated with ischemic stroke has been confirmed in numerous further clinical and preclinical studies (see [8] for a review). A prolonged excess of glucocorticoids is believed to aggravate ischemic brain injury via the unwarranted activation of their receptors and increase the vulnerability of neurons in selected brain regions to neurodegeneration and demise. Disturbances in the HPA axis regulation may lead to a predisposition to stress-induced mental diseases because of a violation in the feedback system controlled by glucocorticoids and pathological alterations in the brain corticosteroid receptors [23,24,25]. The hippocampus and FC modulate the HPA axis, and this control is associated with signaling through the mineralocorticoid and glucocorticoid receptors highly expressed in these regions of the limbic system [26,27]. A close association of HPA axis dysfunction with pro-inflammatory events has been postulated [28,29] and confirmed both in experimental [30,31,32] and clinical studies [33,34,35,36,37]. 

Stroke is a severe neurological pathology characterized by high mortality. In addition, stroke survivors often suffer from delayed post-stroke depressive disorders and cognitive impairments. Importantly in this regard, post-stroke brain damage is not restricted to the infarction area. Nonischemized vulnerable brain regions may be secondarily damaged, and this damage is distant from the primary infarction and postponed in time. The focal damage common for ischemic stroke in the middle cerebral artery circulation is localized in the cerebral cortex and/or striatum, whereas secondary changes are observed in a number of remote areas and first and foremost in the hippocampus which is selectively vulnerable to different kinds of stress [38,39]. As the hippocampus controls both cognitive and emotional functions, the key mechanisms of dementia and depression are associated with its dysfunction. Our hypothesis on the distant hippocampal damage after focal brain injury associates this damage with the pathogenesis of delayed post-stroke cognitive and psychiatric disturbances [9,10]. The hypothesis assumes that glucocorticoids excessively secreted after a focal brain damage interact with their receptors in the hippocampus and induce signal transduction pathways which result in neuroinflammation and subsequent hippocampal neurodegeneration, as well as disturbances in neurogenesis [40]. This may be particularly important for patients with anomalous stress responses due to a dysfunction of the HPA axis. The hippocampus is selectively vulnerable to external factors and responds to glucocorticoid excess by increased cytokine secretion [41]. In addition to cognitive and emotional disorders, stroke may trigger epileptogenesis, leading to epilepsy which is frequently comorbid with depression and cognitive disturbances. We suggest that it is the remote hippocampal damage associated with HPA axis dysregulation that is a shared key link of these comorbidities [8,9,42]. An excess of glucocorticoids, the dysfunction of their receptors, and their progress in neuroinflammatory processes result in hippocampal neuronal loss and the shaping of aberrant neural networks. Though associations between brain ischemia and glucocorticoid-dependent hippocampal damage have been reported in a number of rodent experiments [43,44,45], this hypothesis should be further verified by more experimental and, particularly, clinical data.

It should be noted that the hippocampus and prefrontal cortex are central structures of the cortico–limbic network associated with both cognitive processes (e.g., learning and memory) and the control of emotions [46], whereas their dysconnectivity may underlie different brain diseases [47]. This may explain why both in our previous [15] and present study, we found similar trends to the increased corticosterone accumulation both in the hippocampus and FC of the MCAO-KM rats. Importantly, the accumulation of corticosterone and Il1β in the hippocampus and FC was not only in the injured but also in the contralateral hemisphere, which substantiates the hypothesis of potential distant damage.

Transient MCAO in rodents is one of most common surgical approaches to model ischemic stroke and is believed to be clinically relevant. The most frequent surgery is the insertion of a monofilament to occlude the middle cerebral artery. The classics of surgical approaches using intraluminal filaments for MCAO are the methods of Koizumi et al. [11] and Longa et al. [12] which are employed in preclinical studies worldwide. At present, groups using either of these models regard them as comparable, similar enough to discuss the data taking into account the duration of ischemia at best but not the details of the surgery (the MCAO-KM or MCAO-LM). However, it is well-known that “the devil is in the details”. Indeed, in the MCAO-KM, a monofilament is inserted through the common carotid artery, whereas in the MCAO-LM, it is through the external carotid artery. Critical surgery-specific differences were found in these two MCAO models in mice, including significantly greater reperfusion in the MCAO-LM [14] and a greater and more robust inflammatory response in the MCAO-LM [13]. As the MCAO-KM or MCAO-LM in rats, similar to the respective MCAO models in mice, are presently used randomly, and, most importantly, the data are compared without taking into account the specific features of each model, we have recently studied the early post-stroke period in the MCAO-KM and MCAO-LM in rats and found that, though the infarct volumes and neurological deficits were similar in both models, the MCAO-KM rats demonstrated a greater secretion and accumulation in the hippocampus and FC of corticosterone and IL1β [15]. In the present study, we confirmed our previous data showing a lack of difference between the models in terms of neurological deficits and body mass changes during the first two weeks after the surgery (Figure 2). Furthermore, we demonstrated that 3 months after the surgery, when the concentrations of corticosterone and pro-inflammatory cytokines did not differ from the respective control levels (Table S1), their significant accumulation in the hippocampus and FC of the MCAO-KM (but not the MCAO-LM) animals persisted. The accumulation of corticosterone was evident in both the ipsilateral and contralateral hippocampus equally (Figure 3). IL1β accumulated in the ipsilateral hemisphere on Day 3 [15] but expanded with time to the contralateral hemisphere. The levels of IL1β, an important pro-inflammatory cytokine, were dramatically augmented in both the ipsi- and contralateral hippocampus and FC (Figure 4), whereas the most expressed increase in TNFα levels was found in the ipsilateral hippocampus and FC (Figure 5). Thus, 3 months after MCAO, the difference in the accumulation of corticosterone and cytokines in the hippocampus and FC between the MCAO-KM and MCAO-LM became even more evident 3 days after the surgery [15]. This suggests that this accumulation in the MCAO-KM rats is prolonged and may affect the functioning of these structures and limbic networks in general. The bilateral accumulation of corticosterone early after the MCAO-KM suggested potential damage to the limbic structures of both the injured and “healthy” hemispheres, whereas the bilateral accumulation of both corticosterone and IL1β 3 months after the surgery suggests a steady long-lasting pro-inflammatory trend in the hippocampus and FC and a potential prerequisite of neuronal damage.

It has been shown that transient MCAO induces secondary damages in the hippocampus that is remote from the primary ischemic regions. Focal cortical infarction caused glial activation and neuronal apoptosis in the ipsilateral nonischemic hippocampus, potentially contributing to delayed cognitive deficits [48]. MCAO induced accumulation of hyperphosphorylated tau and the concurrent dephosphorylation of glycogen synthase kinase-3OI (protein kinase involved in NMDA receptor-mediated tau hyperphosphorylation) at Ser 9 in the ipsilateral hippocampus [49]. The secondary brain damage is realized via inflammatory pathways. [(11)C]PK11195 PET detected neuroinflammation in the infarct core as well as in the peri-infarct regions of rats, with both its extent and location independent of the infarct size [50]. A volumetric MRI and 1H MRS study of the hippocampus in unilateral MCAO patients revealed a relationship between hippocampal secondary damage and cognitive disorder following a stroke [51]. The volume and myo-inositol/creatine ratio significantly increased in the hippocampus ipsilateral to occluded middle cerebral artery in stroke patients compared with values in the contralateral hippocampus or healthy control. Importantly, a reduced n-acetyl aspartate/creatine ratio was also observed in the contralateral hippocampus [51]. The delayed changes in the hippocampus and FC induced by focal brain ischemia in MCAO models studied in our experiments may reflect secondary brain damage following the primary injury. According to our hypothesis [8,9,10], we can expect both unilateral and bilateral alterations. Unilateral alterations are more closely associated with the primary injury. Though remote from the infarct core, they are in the same hemisphere. Bilateral changes may be associated with the systemic effects of exterior factors induced by the primary injury. We suggest that activation of the HPA axis is the global systemic factor responsible for distant hippocampal damage after focal brain ischemia, explaining the revealed bilateral changes in the hippocampus. 

As mentioned above, the delayed consequences of ischemic stroke include emotional and cognitive disorders, such as depression and memory disturbances. At first glance, the absence of significant changes in the behavior of the MCAO-KM rats is surprising, taking into account the major alterations in the hippocampus and FC, i.e., the accumulation of corticosterone and pro-inflammatory cytokines. However, a deeper analysis shows that the expectations to find principal delayed behavioral changes might be premature. First, as a general consideration, it is difficult (if possible at all) to reproduce human mental disorders in laboratory animals. No animal model is able to fully mimic any mental disease in humans as these illnesses comprise mental and behavioral disturbances exclusively unique to humans. The same is relevant for cognitive tests [52]. A general approach is to reproduce the particular symptoms of diseases in laboratory animals [53], whereas these symptoms in animals are just, to some extent, analogous to human features, and the interpretation of behavioral changes remains, to some extent, vague from a translational perspective. 

Zarruk et al. [53] pointed to a high variability in the results of behavioral MCAO studies depending on the test and type of brain lesion. Moreover, in different studies, behavioral tests are applied to (a) MCAO models with different durations of ischemia and reperfusion up to permanent MCAO models; (b) “classic” MCAO models modified by the authors [54]; (c) modified MCAO-LM with the introduction of a filament into the internal carotid artery [55]; (d) studies with behavioral tests in different periods after MCAO, from acute to delayed effects. Whereas disturbances in cognitive function and depressive-like symptoms seem more reliable in rodents with permanent MCAO, a model much less clinically relevant than MCAO with reperfusion [56,57], respective changes after transient MCAO seem much more unpredictable due to the variability in experimental conditions used in different studies [58]. Furthermore, observation of the behavioral deficits may critically depend on the post-surgery care [59].

In this study, behavioral tests could not reveal the dramatic delayed effects of MCAO in most tests, including tests for emotional state (anxiety and depression-like behavior in the EPM and sucrose preference tests, respectively); though in the FST, the MCAO rats demonstrated an active stress-coping strategy 3 months after surgery. In general, this is in accordance with the reports of other authors who failed to find any substantial long-term consequences in the emotional state of rats after MCAO-KM [60,61]. Increased anxiety was reported in rats subjected to MCAO-LM; however, the authors interpreted this as a sign of anxiety hyperactive behavior in the OFT [62]. A similar effect was revealed in the MCAO-LM rats in the present study; however, this was not confirmed in the EPM. Ryan et al. [59] did not observe the effect of MCAO-LM on anxiety in the EPM up to two weeks after ischemia.

Post-stroke depressive-like behavior may be detected using the sucrose preference test. Some authors were able to induce depressive-like behavior only when subjected animals to several weeks of unpredictable mild stress [60,61] even using potentially “more severe” MCAO-KM. Despite this, another group found “anhedonia” in mild MCAO-LM [55], but such post-stroke depression was observed in only 56.25% of surviving animals. In the present study, we did not find any effect of MCAO in either the sucrose preference or forced swim tests. Moreover, the MCAO-KM and MCAO-LM rats exhibited increased swimming activity and/or active strategies to cope with the water immersion stress. We have previously shown that induced hyperactivity in a model of agitated depression induced by bulbectomy may lead to the misinterpretation of animal behavior in tests for anxiety and depression [63].

MCAO did not affect the spontaneous alternation behavior in a Y-maze indicative of short-term (working) memory. However, some behavioral differences between the MCAO-KM and MCAO-LM were found. First, in the OFT, the rats in the MCAO-LM group traveled a longer distance with higher velocity compared with the respective sham-operated group (Figure S2G,H), whereas the behavior of the MCAO-KM rats was similar to the respective shams. According to the performance of the passive avoidance test, we could conclude that long-term memory was impaired in the MCAO-LM group only (Figure 7C). Indeed, Zvejniece et al. [58] found memory impairment in a passive avoidance test only in the 120 min MCAO-LM group (not the 90 min MCAO group) 6 days after the acquisition trial, whereas [64] reported impaired passive avoidance response 1 month after 90 min of MCAO-LM. Interestingly, Ryan et al. [59] observed worse learning capacities but not memory in rats subjected to MCAO-LM in the Morris water maze 2 months after surgery. Despite this, Bouët et al. [65] showed that after 3 h of MCAO, mice showed normal spatial learning abilities in the Morris water maze test, but they displayed learning deficits measured by the passive avoidance test. The authors also showed that this latter deficit was significantly correlated with both cortical and striatal damage. However, this cannot explain our data as the infarct volume did not differ between the MCAO-LM and MCAO-KM [15]. We also should take into account a significant dispersion in the reaction of rats in the passive avoidance test used (Figure 7C). Furthermore, 7 of the 13 SHAM-KM rats and 4 of the 8 MCAO-KM rats exhibited maximal latency of entry into the dark compartment, whereas 7 of the 10 SHAM-LM rats and 2 of the 9 MCAO-LM rats exhibited the same maximal latency. It seems that the effect of ischemia on long-term memory was more expressed in the MCAO-LM; but this difference was not supported by statistical analysis (two-tailed Fisher exact *p* = 0.06). Obviously, the results of the passive avoidance test should be repeated to make them valid and, probably, more tests for learning and memory, such as the Morris water maze, should be applied to compare different MCAO models a long time after surgery.

## 5. Conclusions

The differences between the MCAO-KM and MCAO-LM models which we detected during the first days after MCAO in the previous study [15] became even more obvious after 3 months. The MCAO-KM (but not the MCAO-LM) group showed significant accumulation of corticosterone and IL1β in both the ipsilateral and contralateral hippocampus and FC. Accumulation of TNFα was detected only in the ipsilateral hippocampus and FC in the MCAO-KM group. Thus, unlike the MCAO-LM, the MCAO-KM model may predispose the hippocampus and FC of rats to long-lasting bilateral corticosterone-dependent distant neuroinflammatory damage. The MCAO-KM and MCAO-LM are used as alternatives in preclinical studies. However, the revealed differences between the two MCAO models, particularly associated with the continuing increase in corticosterone and proinflammatory cytokine accumulation in the limbic structures in the MCAO-KM, should not be neglected in the planning of preclinical experiments and the interpretation and translation of received results. This is especially vital in studies of approaches to prevent delayed post-stroke disturbances.

## 6. Limitations

The main limitation of this study is the lack of the pathomorphological investigation of the hippocampi of rats. Unfortunately, it was impossible to do this with the hippocampal material from the experimental animals as the hippocampi and FC of both hemispheres were entirely used for biochemical analyses. Second, 3 months after occlusion, it is impossible to correctly assess the initial volume of infarction due to the replacement of neurons with proliferating glia. In this study, the presence of the lesions was judged according to the development of neurological deficit in the early post-stroke period. However, the infarct volumes in the MCAO-KM and MCAO-LM 3 days after surgery were reported in our previous study [15]. Third, as the evaluation of age- or sex-related differences in the MCAO models was out of the scope of this study, only young male Wistar rats were used. Finally, in all experiments with significant animal lethality, we are facing so called “survivorship bias”, i.e., a type of sample selection bias that occurs when a data set only considers “surviving” or existing observations and fails to consider observations that already ceased to exist. The high mortality, usual for the MCAO models used [13,14,15], could induce survivorship bias potentially affecting the conclusions. Indeed, we do not know anything about the corticosterone levels in the rats of the MCAO groups who died within the course of the experiment. Though we are unable to overcome this bias using the experimental design employed, it should be kept in mind when the data are analyzed.

## Data Availability

Data available on the demand.

## Supplementary Materials

The following supporting information can be downloaded at: https://www.mdpi.com/article/10.3390/biomedicines10123119/s1, Table S1. Blood levels of corticosterone (CS), ACTH, IL1β, TNFα and IL6 in MCAO-KM and MCAO-LM groups 3 month after surgery. Table S2. Brain levels of corticosterone (CS), IL1β, TNFα and IL6 in MCAO-KM and MCAO-LM groups 3 month after surgery. Figure S1. IL6 levels in the hippocampus (A,B) and FC (C,D) in MCAO-KM and MCAO-LM groups 3 months after surgery. (A) Ipsilateral hippocampus. (B) Contralateral hippocampus. (C) Ipsilateral FC. (D) Contralateral FC. (A-D) IL6, pg/g tissue. Figure S2. Effects of focal brain ischemia on behavior of MCAO-KM and MCAO-LM groups in the open field test 3 months after surgery. (A) Total distance traveled during the testing. (B,C). Distance traveled in the central and peripheral zones of the arena, respectively. (D) Total number of rearing. (E) Exploratory rearing. (F) Climbing. (G) Velocity. (H) Ratio of distance traveled in the central and peripheral zones of the arena. (I) Number of defecation boli. * *p* < 0.05, ** *p* < 0.01 and *** *p* < 0.001 (Mann-Whitney U-test). Figure S3. Effects of focal brain ischemia on behavior of MCAO-KM and MCAO-LM groups in the elevated plus maze 3 months after surgery. (A,B) Entries into the closed (CA) or open arms (OA). (C,D) Time spent in the closed or open arms, respectively.

## Abbreviations

ACTH	adrenocorticotropic hormone
ELISA	enzyme-linked immunosorbent assay
EPM	elevated plus maze
FC	frontal cortex
FST	forced swim test
HPA	hypothalamic–pituitary–adrenal
IL1β	interleukin 1beta
IL6	interleukin 6
MCAO	middle cerebral artery occlusion
MCAO-KM	middle cerebral artery occlusion, Koizumi et al. model
MCAO-LM	middle cerebral artery occlusion, Longa et al. model
OFT	open field test
TNFα	tumor necrosis factor-alpha
