# Supplementary material for: A Comparative Study of Two Models of Intraluminal Filament Middle Cerebral Artery Occlusion in Rats: Long-Lasting Accumulation of Corticosterone and Interleukins in the Hippocampus and Frontal Cortex in Koizumi Model
Source: Biomedicines. 2022 Dec 2;10(12):3119. doi: 10.3390/biomedicines10123119 (PMC9775077; doi:10.3390/biomedicines10123119)
Supplement: Supplementary file 1 [file biomedicines-10-03119-s001.zip › biomedicines-1918868-supplementary.pdf]

Table S1. Blood levels of corticosterone (CS), ACTH, IL1β, TNFα and IL6 in MCAO-KM and MCAO-LM groups 3 month after surgery

Groups	SHAM-KM n=12	MCAO-KM n=8	SHAM-LM n=10	MCAO-LM n=9
**CS, nmol/ml**	1.33±0.08	1.39±0.15	1.13±0.17	0.89±0.12◊
**ACTH, ng/ml**	4.44±0.61	4.87±1.07	3.06±0.39	4.79±0.59
**IL1β,pg/ml**	79.96±18.94	77.38±28.64	70.15±17.62	74.14±20.51
**TNFα, pg/ml**	10.75±3.92	7.00±2.14	10.45±3.18	9.28±3.24
**IL6,pg/ml**	73.14±13.21	72.25±7.99	57.10±12.33	79.11±9.92

◊ - $p< 0.1$ MCAO-KM vs. MCAO-LM.

Table S2. Brain levels of corticosterone (CS), IL1β, TNFα and IL6 in MCAO-KM and MCAO-LM groups 3 month after surgery

Groups	SHAM-KM n=12	MCAO-KM n=8	SHAM-LM n=10	MCAO-LM n=9
		**Ipsilateral hippocampus**		
**CS, pmol/g tissue**	85.6±7.7	169.0±35.7*	144.2±30.8	119.9±10.6
**IL1β, pg/g tissue**	61.0±16.8	562.4±44.6*	416.7±60.9	150.3±30.3*◊
**TNFα, pg/g tissue**	355,3±26.7	657.9±55.4**	440.3±32.4	378.0±29.4◊◊
**IL6, ng/g tissue**	2.49±0.13	3.01±0.38	2.93±0.20	2.65±0.32
		**Contralateral hippocampus**		
**CS, pmol/g tissue**	71.0±7.8	205.1±39.4**	151.9±21.6	100.2±20.2◊
**IL1β, pg/g tissue**	80.0±30.7	643.9±63.6*	202.2±22.3	82.4±19.1◊
**TNFα, pg/g tissue**	305.3±9.1	374.6±19.9**	317.9±5.6	305.7±10.4◊
**IL6, ng/g tissue**	2.38±0.17	2.52±0.16	2.74±0.24	2.32±0.17
		**Ipsilateral FC**		
**CS, pmol/g tissue**	125.5±4.5	169.9±22.3	133.3±17.3	100.8±11.0◊
**IL1β, pg/g tissue**	129.6±41.4	726.8±51.2**	382.5±17.62	171.0±30.7*◊◊
**TNFα, pg/g tissue**	228.4±16.8	471.6±53.6**	205.7±8.0	208.5±14.7◊◊

**IL6, ng/g tissue**	2.37±0.15	2.71±0.31	2.53±0.18	2.19±0.18
		**Contralateral FC**		
**CS, pmol/g tissue**	123.3±9.6	167.7±20.0	146.2±18.8	106.5±14.1
**IL1β, pg/g tissue**	313.1±70.4	525.0±52.6**	254.1±67.1	42.5±29.8*◊◊
**TNFα, pg/g tissue**	327.4±50.4	268.3±18.2	236.1±18.0	213.8±21.5
**IL6, ng/g tissue**	2.18±0.11	2.43±0.34	2.49±0.18	2.19±0.17

**,◊◊ $p < 0.01$ and *,◊ $p < 0.05$ for respective sham-operated groups vs MCAO (asterisk) or MCAO-KM vs MCAO-LM (rhombus).

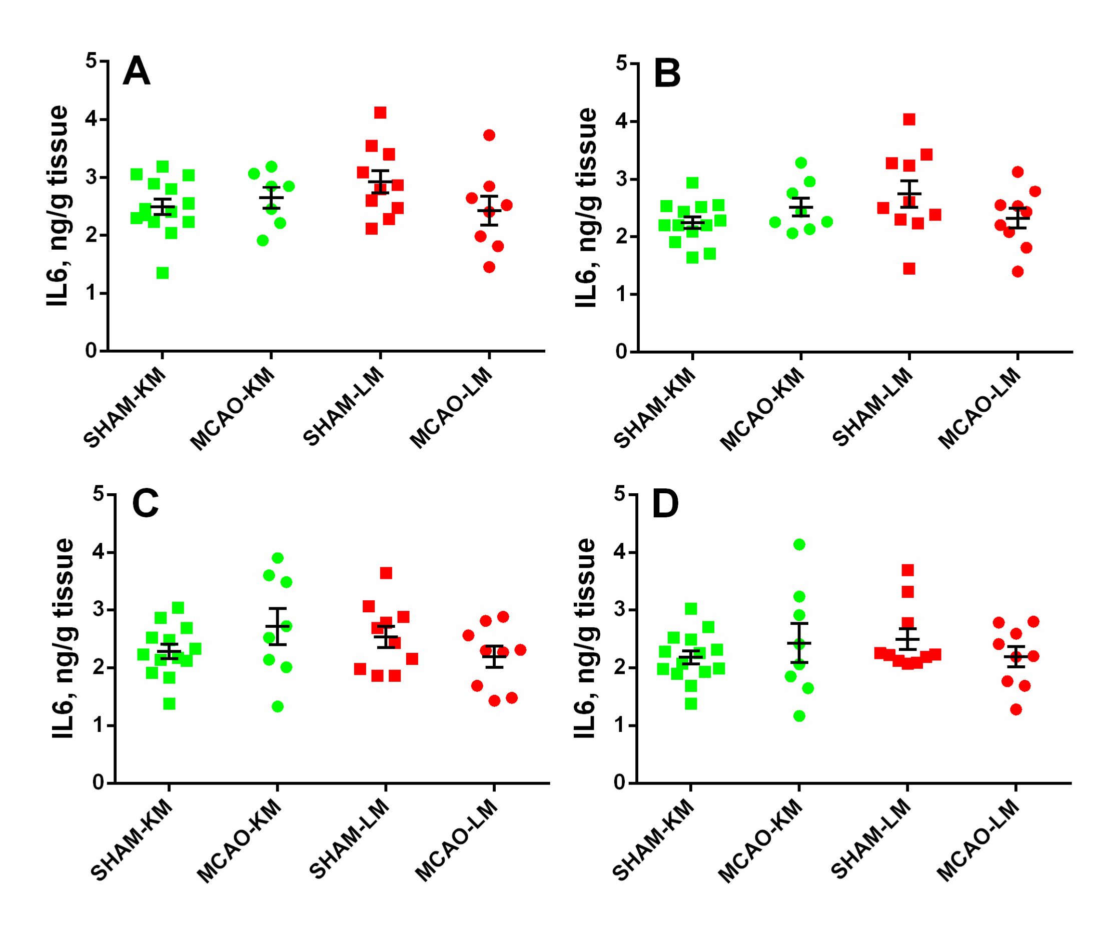

Figure S1. IL6 levels in the hippocampus (A, B) and FC (C, D) in MCAO-KM and MCAO-LM groups 3 months after surgery. (A) Ipsilateral hippocampus. (B) Contralateral hippocampus. (C) Ipsilateral FC. (D) Contralateral FC. (A-D) IL6, pg/g tissue.

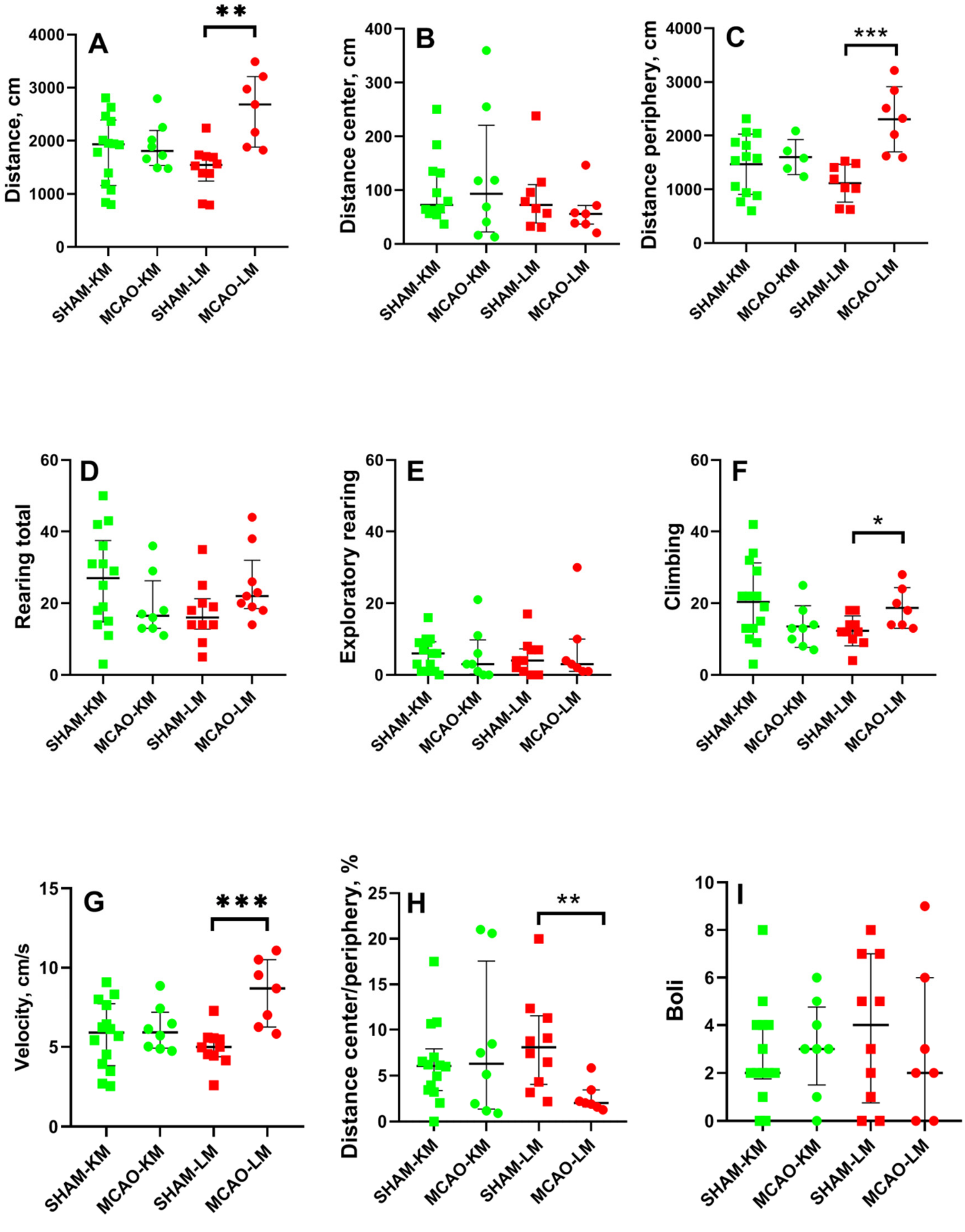

Figure S2. Effects of focal brain ischemia on behavior of MCAO-KM and MCAO-LM groups in the open field test 3 months after surgery. (A) Total distance traveled during the testing. (B) and (C). Distance traveled in the central and peripheral zones of the arena, respectively. (D) Total number of rearing. (E) Exploratory rearing. (F) Climbing. (G) Velocity. (H) Ratio of distance traveled in the central and peripheral zones of the arena. (I) Number of defecation boli. * - $p < 0.05$, ** - $p < 0.01$ and *** $p < 0.001$ (Mann-Whitney U-test).

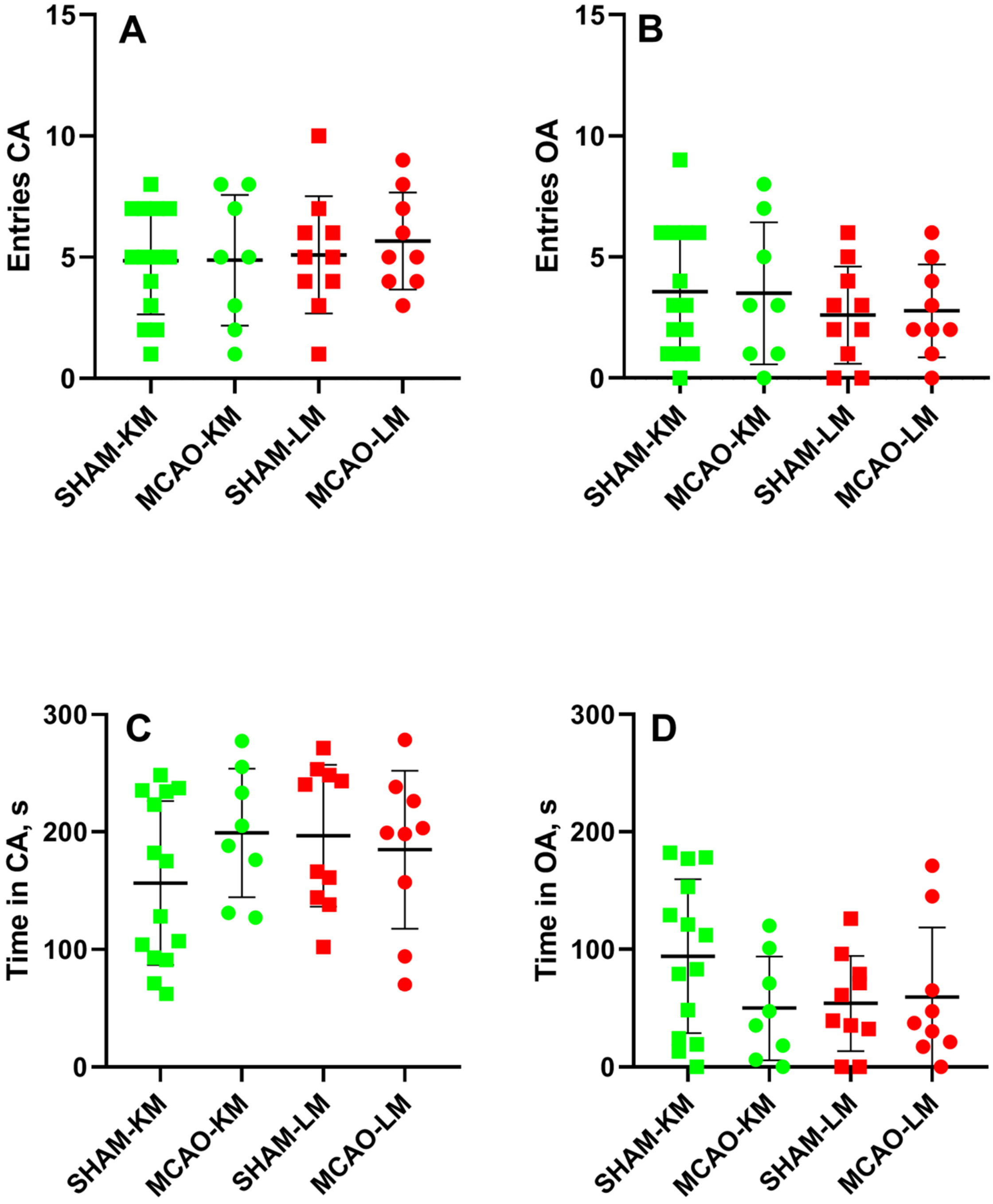

Figure S3. Effects of focal brain ischemia on behavior of MCAO-KM and MCAO-LM groups in the elevated plus maze 3 months after surgery. (A) and (B) Entries into the closed (CA) or open arms (OA). (C) and (D) Time spent in the closed or open arms, respectively.
